# Carbon catabolite repression in pectin digestion by the phytopathogen *Dickeya dadantii*

**DOI:** 10.1016/j.jbc.2021.101446

**Published:** 2021-11-23

**Authors:** Shiny Martis B, Michel Droux, William Nasser, Sylvie Reverchon, Sam Meyer

**Affiliations:** Laboratoire de Microbiologie, Adaptation et Pathogénie, Université de Lyon, INSA Lyon, Université Lyon 1, CNRS UMR 5240, Villeurbanne, France

**Keywords:** metabolism, gene regulation, bacteria, pathogenicity, plant, systems biology, simulation, pectin, carbon catabolite repression, CRP, cAMP receptor protein, KDG, 2-keto-3-deoxygluconate, Pel, endo-pectate lyase, PGA, polygalacturonate

## Abstract

The catabolism of pectin from plant cell walls plays a crucial role in the virulence of the phytopathogen *Dickeya dadantii*. In particular, the timely expression of *pel* genes encoding major pectate lyases is essential to circumvent the plant defense systems and induce massive pectinolytic activity during the maceration phase. Previous studies identified the role of a positive feedback loop specific to the pectin-degradation pathway, whereas the precise signals controlling the dynamics of pectate lyase expression were unclear. Here, we show that the latter is controlled by a metabolic switch involving both glucose and pectin. We measured the HPLC concentration profiles of the key metabolites related to these two sources of carbon, cAMP and 2-keto-3-deoxygluconate, and developed a dynamic and quantitative model of the process integrating the associated regulators, cAMP receptor protein and KdgR. The model describes the regulatory events occurring at the promoters of two major *pel* genes, *pelE* and *pelD*. It highlights that their activity is controlled by a mechanism of carbon catabolite repression, which directly controls the virulence of *D. dadantii*. The model also shows that quantitative differences in the binding properties of common regulators at these two promoters resulted in a qualitatively different role of *pelD* and *pelE* in the metabolic switch, and also likely in conditions of infection, justifying their evolutionary conservation as separate genes in this species.

The expression of virulence genes is known to respond to metabolic changes in many pathogenic species, including *Salmonella enterica* (1), *Vibrio cholerae* (2), *Helicobacter pylori* (3), *Pseudomonas aeruginosa* (4), *Escherichia coli*, and *Shigella* spp (5), revealing a tight interconnection between virulence and metabolic functions (6). This link is particularly conspicuous in the phytopathogen *Dickeya dadantii*, a soft rotting Gram-negative bacterium that attacks a wide range of plant species, including many crops of economical importance (7). In a first asymptomatic phase of infection, *D. dadantii* colonizes the intercellular space (apoplast) where it grows on available simple sugars produced by the plant. The symptomatic phase, maceration, is characterized by the degradation of pectin, a complex sugar present in the plant cell walls, resulting in soft rot, the main visible symptom (8). The success of the infection process crucially depends on *D. dadantii*’s ability to switch from glucose to pectin catabolism in a timely and efficient manner, to overcome attacks by the plant’s defense systems (9).

The depolymerization of the pectin polysaccharide is achieved by a variety of pectinases. Among these, endo-pectate lyases (Pels) are known to play a prominent role and considered as major virulence factors (8), especially those encoded by the *pelD* and *pelE* paralogous genes (10). In the presence of pectin, the expression of *pel* genes is induced *via* a positive feedback loop: the binding of the main repressor KdgR at their promoters is relieved in presence of its cofactor 2-keto-3-deoxygluconate (KDG) itself resulting from the degradation of pectin by Pel enzymes (11). This nonlinear mechanism has been formerly proposed to explain the strong boost in *pel* expression required for a fast switch to the maceration phase (12). However, the culture experiments showed that pectin is degraded early during bacterial growth, whereas *pel* expression peaks only hours later, close to the transition to stationary phase (12). In this paper, we propose a new model for this regulatory loop, where the expression of pel genes is triggered not by the degradation of pectin alone, but rather by the shift in metabolic uptake from glucose to pectin. We show that this shift is controlled by a carbon catabolite repression mechanism, that is, the selective uptake of a preferred carbon source (glucose) by repression of the catabolism of other sources (here pectin). It presents many similarities but also interesting differences with the classical glucose/lactose example in *E. coli*, in particular regarding dynamical properties required for a successful infection (13).

Although the regulation of pectin degrading *pel* genes involves the combined action of at least a dozen different transcription factors and nucleoid-associated proteins (8), the strongest of these regulators monitored in the cell are KdgR and cAMP receptor protein (CRP) (8). Because the latter is also the main catabolite-activator protein controlling the glucose metabolic switch in *E. Coli* (14), we hypothesized that the combined action of these two regulators could explain the time course of *pel* expression through variations in the concentration of their respective cofactors, KDG and cAMP. To validate this hypothesis, we used a highly sensitive method to measure the concentrations of these metabolites, based on a derivatization of the compounds followed by HPLC quantification. Based on these data, we present a quantitative dynamical model of the regulation pathway. The binding affinities of both regulators with their cofactors and *pel* operators were formerly measured (12, 15) as well as other key parameters of the system, requiring only five adjustable parameters in the model. The latter explains the dynamics of Pel production during bacterial growth when both glucose and pectin are available as well as the specific role of the recently diverged paralogous genes *pelE* and *pelD* in pectinolysis (10), explaining why this divergence could constitute an evolutionary advantage for pathogenesis.

## Results

### Pectin degradation and KDG concentration peak occur before Pel production boost

**Figure 1:**
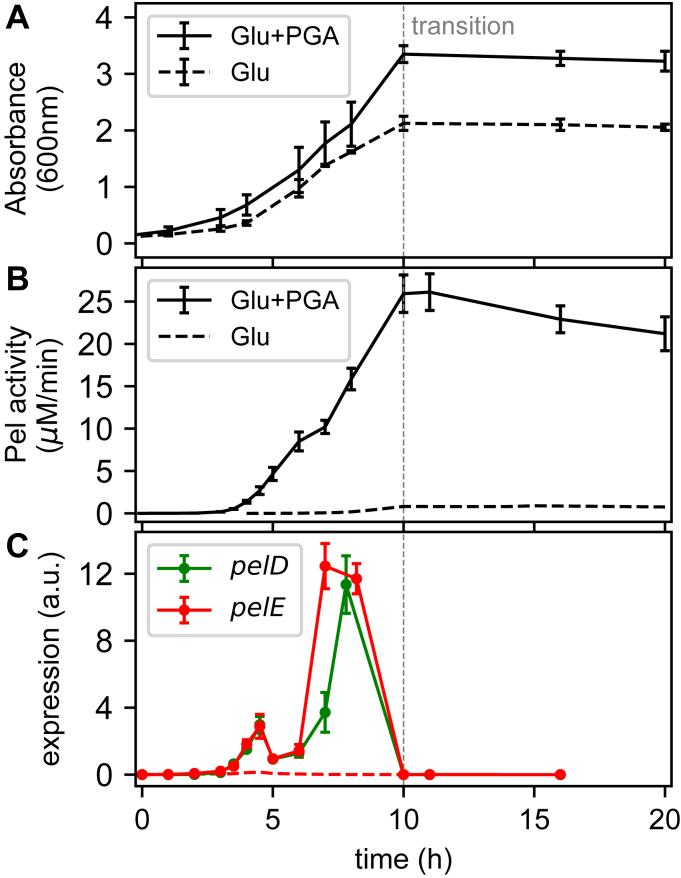
**Dynamics of bacterial growth and Pel production.***A,* growth curves of *Dickeya dadantii* in M63 minimal medium supplemented with glucose (*dashed*) or glucose+PGA (*solid*). The transition time is indicated by a *gray vertical dashed line* and is used as a reference timepoint throughout the article. *B*, total Pel enzymatic activity measured in the extracellular space along bacterial growth in glucose (*dashed*) or glucose+PGA (*solid*) media. PGA induces Pel activity by a factor of around 30. *C*, *pelD* and *pelE* expression measured by qRT-PCR along bacterial growth in glucose+PGA medium (*green* and *red solid lines*, respectively). *pelE* expression levels are also shown in the absence of PGA (*dashed red line*). All the error bars represent 95% confidence intervals obtained from two biological replicates. Pel, endo-pectate lyase; PGA, polygalacturonate.

**Figure 2:**
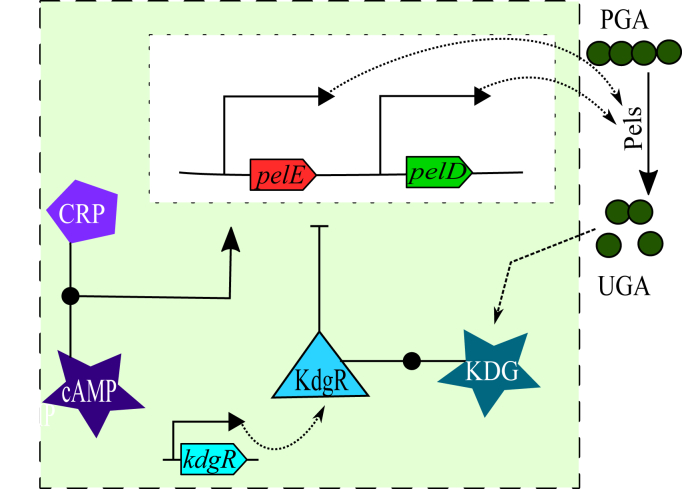
**Diagram of the components and reactions in the proposed dynamical model of pectin catabolism.** The two metabolites, cAMP and KDG, indirectly activate the expression of *pel* genes by binding their associated regulators: cAMP allows CRP to bind and activate the promoters, whereas KDG relieves KdgR repression, respectively. Pel enzymes are instantaneously exported and degrade PGA (pectin) into UGA (unsaturated galacturonides), which is imported and converted into KDG, triggering a positive feedback loop. KdgR is synthesized at a constant rate. CRP, cAMP receptor protein; KDG, 2-keto-3-deoxygluconate; Pel, endo-pectate lyase; PGA, polygalacturonate.

**Figure 3:**
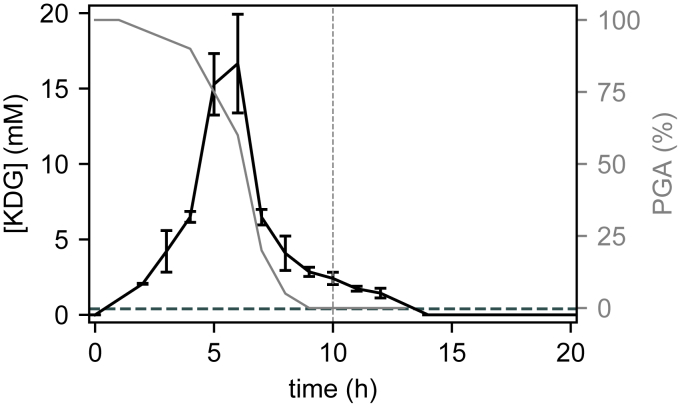
**Time course of PGA degradation (*gray line*, data from** (12)**) and intracellular KDG concentration measured by HPLC (*black solid line*, see**Experimental procedures**).** The *horizontal dashed line* (0.4 mM) indicates the KDG-KdgR affinity: above this level, the repressor is bound by KDG, repression is relieved, and *pel* expression is strongly induced. The transition time is indicated by a *gray vertical dashed line*. The error bars represent 95% confidence intervals obtained from two biological replicates. KDG, 2-keto-3-deoxygluconate; Pel, endo-pectate lyase; PGA, polygalacturonate.

The production of pectate lyases was monitored during *D. dadantii* growth in minimal medium supplemented with glucose or glucose+polygalacturonate (PGA), a simple form of pectin (Fig. 1*A*). The latter acts as an inducer, boosting the production of enzymes by a factor of around 30 (Fig. 1*B*), which was previously attributed to the indirect activation of *pel* genes by the metabolite KDG resulting from PGA degradation (11, 12, 15): when KDG binds the regulator KdgR, it relieves transcriptional repression by the latter, thus triggering a positive feedback loop of pectin degradation (Fig. 2). However, this scenario is challenged by the dynamics of PGA degradation (Fig. 3, gray line, data from (12)), which occurs quickly, after only around 5 h of growth, long before most Pel enzymes are produced (close to transition). To define this delay with more precision, we directly measured the expression pattern of two major *pel* genes (*pelD* and *pelE*) by qRT-PCR, which exhibits a much higher time resolution (a few minutes) than the Pel activity assay, whose slow kinetics is because of the long lifetime of Pel enzymes (around 24 h) (12). Indeed, both *pelD* and *pelE* exhibit a sharp expression peak (after very low initial levels), and it does not occur before around 7 to 8 h of growth (Fig. 1*C*). This assay confirms that the activation of Pel by PGA occurs at the transcriptional level, because the levels of *pel* expression in glucose medium are very low (red dashed line). To explain this delay between PGA degradation and Pel production, a very slow import of the extracellular pectin degradation products (unsaturated galacturonides) and/or subsequent intracellular conversion into KDG inducer was invoked (Fig. 2, the detailed pathway of pectin degradation is shown in Fig. S1); yet this scenario seems unrealistic, because depolymerization rather than import is thought to be the rate-limiting step of this pathway and because such a slow kinetics would be a major obstacle for the fast activation of *pel* genes by pectin required for efficient plant infection (9).

Resolving the discrepancy required a direct measurement of intracellular KDG concentration. This was achieved using a new method based on the derivatization of the carbonyl group of the molecule with o-phenylenediamine and subsequent HPLC quantification (details in Experimental procedures). Elution with a nonlinear increasing gradient of TFA and acetonitrile allows separating KDG from its close structural analogs, as described in (16). The samples collected at regular time intervals clearly indicate that the KDG-inducer peak occurs almost simultaneously with PGA degradation, after around 6 h of growth (Fig. 3), confirming that the import of oligogalacturonides and further degradation steps into KDG are extremely fast and cannot be the limiting steps for strong *pel* expression. Most strikingly, the lifetime of KDG is also quite short in the cell, because the concentration has already dropped to half its value 1 h after its peak time (the growth rate is by far insufficient to explain this decrease through a dilution effect, because the absorbance increases by only around 30% in the same time interval, see Fig. 1*A*). This profile should be compared with the expression profile of *pelD* and *pelE* (Fig. 1*C*), which peak almost entirely after the concentration of the KDG inducer has dropped. An alternate or additional regulatory mechanism is thus necessary involved.

### Pel production time is mainly controlled by cAMP accumulation

*pel* genes exhibit a particularly complex regulation, probably optimized for the specific requirements of virulence where any error in their expression time and strength results in the detection, attack, and ultimately, destruction of the pathogen by the host’s defense systems (9). This regulation involves global transcription factors (CRP) and nucleoid-associated proteins (Fis, H-NS, and IHF), which act in combination with DNA supercoiling (17), as well as more classical regulators of the pectin-catabolic pathway (KdgR, PecT, PecS, and MfbR) (8). Several of these regulators might contribute in relating *pel* expression to the metabolic state of the cell, such as the Fis repressor that is mostly present in early exponential phase or DNA supercoiling (17). However, the latters’ quantitative effect on *pel* expression is known to be significantly milder than that of CRP and KdgR (17, 18), which are considered as the main regulators and whose binding and regulatory properties on several *pel* promoters were therefore analyzed with much detail, both *in vitro* (15, 18) and *in vivo* (11, 19). We took advantage of this accumulated knowledge to develop a quantitative modeling of the regulatory dynamics of the system.

**Figure 4:**
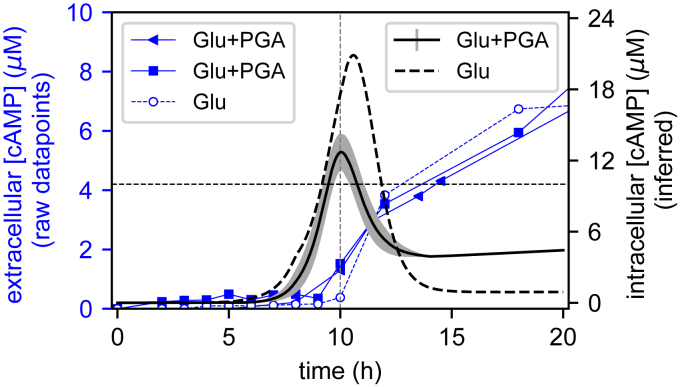
**Time course of measured extracellular cAMP concentration (*blue datapoints*) and inferred intracellular cAMP concentration profile (*black lines*).** The *solid lines* correspond to the cultures grown in glucose+PGA medium: datapoints from two biological replicates are indicated by *squares* and *triangles*, and the *shaded gray area* around the *continuous line* indicates one SD of the intracellular concentration. The datapoints obtained in the presence of glucose are indicated with *dashed lines* (*empty circles* for raw datapoints and *dashed black line* for intracellular concentration). The *dashed horizontal line* (10 μM) indicates the cAMP-CRP binding affinity (43): above this level, cAMP binds CRP, and *pel* expression is activated. CRP, cAMP receptor protein; Pel, endo-pectate lyase; PGA, polygalacturonate.

The first step consisted in quantifying the second main contribution of the regulatory signal, that is, the concentration of the metabolite cAMP, which triggers a conformational change in CRP that allows it to bind most of its operator sites and play its activating role (Fig. 2 and (18)). This quantification was performed by HPLC after derivatization using 2-chloroacetaldehyde for fluorescence detection (20) (see Experimental procedures). The samples were collected at regular time intervals during growth, and cAMP concentrations were quantified in the extracellular medium. Internal cAMP concentrations were then inferred from these latter by means of a kinetic model of cAMP export and import, as previously proposed (21) (see Supplementary Information, p. S4). The resulting intracellular cAMP concentration profile (Fig. 4) entirely matches previous profiles obtained by both direct (22, 23) and indirect measurements in *E. coli* (21), with a very low level observed during exponential growth in glucose, a sharp peak at the transition to stationary phase, and lower but nonzero concentrations in the latter.

The cAMP concentration time course was monitored during growth on glucose with or without PGA (Fig. 4). The profiles are qualitatively similar, except that the peak is somewhat sharper when glucose is the only sugar source present, as we expected. In both conditions, the binding of a significant fraction of CRP proteins present in the cell (the affinity is indicated as a dashed horizontal line), subsequent promoter binding by the cAMP–CRP complex and Pel enzyme production boost are thus expected to occur around the transition to stationary phase, compatible with observations (Fig. 1, *B* and *C*). In summary, KDG acts as an inducer of Pel production (as visible in the relative amounts of enzymes produced in the two media), whereas the timing of this production is thus rather controlled by cAMP, in contrast with previous hypotheses (12). Note that, for the concentration of PGA used in these experiments, the levels of internal KDG concentration reached in the cell are around 40 times higher than those required to bind KdgR and relieve the repression (0.4 mM, horizontal dashed line in Fig. 3), explaining why the inducing effect can last hours after KDG concentration has started to decrease. In other words, only a tiny fraction of Pels, produced very early, is sufficient to degrade the whole amount of pectin present in our culture medium, whereas the actual peak of Pel production occurs only hours later, when cAMP starts to accumulate in the cell close to the transition to stationary phase.

### Combined regulation of *pel* genes through KDG and cAMP metabolites

We now translate these qualitative observations into a quantitative model, schematized in Figure 2, which explicitly incorporates two *pel* genes with major virulence effect, *pelD* and *pelE*. To facilitate the understanding, we first describe the model without distinguishing these two genes which share the same regulators and produce enzymes of similar activity, leaving the analysis of their differential effects to the next section.

The dynamics of all components (bacterial cell density and concentrations of enzymes, regulators, and metabolites inside/outside the cells) are simulated over time, according to a set of coupled differential equations reflecting standard descriptions of the associated molecular events. The details are given in Experimental procedures, and lists of equations and parameters can be found in Supplementary Information. All binding events are described as thermodynamic equilibria, in particular those between the key metabolites (KDG and cAMP) and the associated regulatory proteins (KdgR and CRP, respectively) and those between the latter and the *pel* promoters. All enzymatic reactions are assumed to follow Michaelis–Menten kinetics. For simplicity, bacterial growth follows a predefined logistic equation, where the maximal growth rate and final density depend linearly on the available nutrients (glucose and/or PGA) (12). Based on these hypotheses, most parameters of the system are known from previous experimental knowledge (Table 1 and Table S2), and the five remaining parameters (Pel production rates and kinetic parameters controlling KDG synthesis and degradation, see Table S3) were numerically adjusted to reproduce the observations of Figures 1 and 3 (see Experimental procedures).

**Table 1:** Experimental regulatory parameter values and expression levels measured and obtained by the model after optimization, for *pelD* and *pelE*

Promoter	*pelD*	*pelE*
KdgR binding affinity (nM)	0.9	10
cAMP-CRP binding affinity (nM)	0.3	0.6
cAMP-CRP activation factor	181	62
PGA induction factor (exp)	330	83
PGA induction factor (model)	324	93

**Figure 5:**
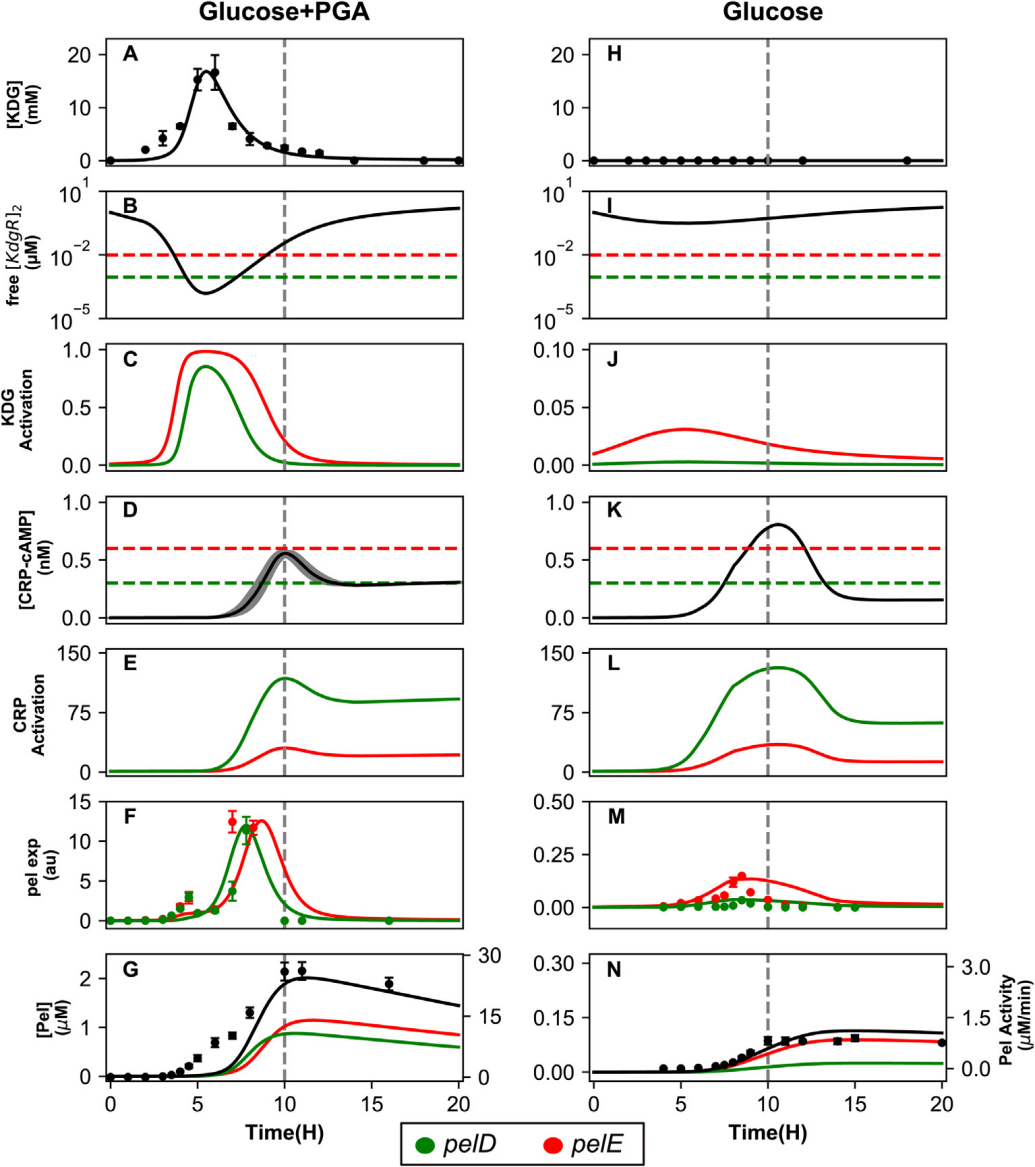
**Modeling of *pel* regulatory pathways and expression in bacteria grown in minimal medium supplemented with glucose+PGA (*left column*) or glucose (*right*).** The model results are shown as *solid lines*, and the experimental data as *dots*. The transition time is shown as a *dashed gray vertical line*. *A*, KDG intracellular concentration superimposed with HPLC direct measurement data exhibiting a peak after around 6 h of growth; *B*, concentration of intracellular free KdgR dimer, with *horizontal dashed lines* showing the binding affinities of the dimer for the *pelE* (*red*) and *pelD* (*green*) promoters, indicating the thresholds of repression; *C*, *pelE* and *pelD* (depicted in *red* and *green*, respectively throughout the figure) activation curves based on KdgR binding, exhibiting a derepression in the middle of exponential phase due to high intracellular levels of KDG; *D*, intracellular concentration of the cAMP–CRP complex inferred from HPLC measurement of cAMP in the medium (Fig. 4); the *gray area* is the 95% confidence interval (from two biological replicates), and the *dashed horizontal lines* indicate the binding affinities for *pelD/E* promoters; *E*, *pel* activation curves based on cAMP-CRP binding, with a boost occuring at transition; *F*, *pel* expression time course based on the combined regulation by KdgR and CRP, superimposed with qRT-PCR datapoints peaking around 8 h; *G*, extracellular concentration of Pel enzymes, either from individual genes (*red* and *green*) or the combination of both (*black*), superimposed with the measurements of enzymatic activity (*dots*) reflecting total Pel enzymes, including those not considered in our modeling. *H*–*N*, same legends as *A*–*G*: in the absence of PGA, the dilution of KdgR during growth is sufficient to partly relieve *pelE* repression, whereas cAMP induces the expression of both *pels* close to transition. The timing and expression levels of both genes are accurately reproduced by the model in both conditions, as well as the inducing effect of PGA. CRP, cAMP receptor protein; KDG, 2-keto-3-deoxygluconate; Pel, endo-pectate lyase; PGA, polygalacturonate.

The essential steps and results of the modeling are illustrated in Figure 5. The KDG profile results from the degradation of PGA by Pel enzymes previously produced and exported (A). Although KdgR is synthesized in the cell at a constant rate, it is mostly bound by KDG when the latter is present in sufficient quantity (over 0.4 mM), resulting in the unbinding of *pel* gene promoters (B) and relieve of KdgR repression (C) compared with cells grown with glucose only (J). The expression of *pel* genes is computed based on a thermodynamic model of transcription (24), with experimental values of KdgR/CRP affinities and activation factors (Table 1). To illustrate the effect of each regulatory signal, we compute activation profiles (Fig. 5, *C*, *E*, *J*, and *L*), representing the gene expression fold-change due to repressor (<1) or activator (>1) binding, and proportional to the statistical weight of the unbound or bound state of the corresponding regulatory protein (here KdgR and CRP), respectively. Because the KdgR–KDG regulatory system involves a positive feedback loop of highly nonlinear behavior, reproducing the measured KDG peak is the most sensitive step in the numerical tuning procedure because it depends on several kinetic parameters of unknown value (Table S3), whereas the regulatory part of the model entirely relies on knowledge-based parameter values.

To keep the modeling as simple as possible, we considered the internal cAMP concentration profile as an input information (Fig. 4). cAMP binds CRP with a measured affinity, assuming a constant concentration of the protein in the cell, resulting in a concentration peak of the cAMP–CRP complex at the transition (Fig. 5*D*). In turn, this complex binds and activates *pel* gene promoters based on measured affinities and activating factors (Table 1 and Fig. 5*E*) (15). The peak in *pel* expression thus results from the combination of the two latter regulatory signals, which are separated in time but exhibit a short overlap after around 7 to 8 h of growth, in relatively good agreement with our qRT-PCR data both in presence and absence of pectin (Fig. 5, *F* and *M*). Similarly, the production level and timing of total Pel enzymes match the experimental profiles, with an approximate 30-fold induction by PGA (Fig. 5, *G* and *N*). Note that the measured Pel enzymatic activity exhibits a slow accumulation already at an earlier time than in the model and matching with early secondary peaks of *pelE/D* expression, close to the peak time of KDG accumulation in the cell (Fig. 5*A*). This discrepancy may reflect several limitations of the model: (1) our simple activation model (Fig. 5*C*) becomes inaccurate at high KDG concentrations; (2) additional regulators modulating *pel* expression are disregarded (see next section); and (3) various other *pel* genes contribute to the overall Pel activity profile.

### Quantitative differences in regulatory sequences underpin qualitatively different roles for paralogous genes

In the modeling, we explicitly considered two separate *pel* genes, *pelD* and *pelE*, which play a major role in virulence as observed from pathogenicity assays (25) and were therefore studied extensively (17, 26). These paralogous genes result from a recent duplication event from a common ancestor present in other *Dickeya* species (10) respond to the same regulators and encode proteins of similar enzymatic activity, yet were shown to play different roles during bacterial growth and plant infection (10). Because the regulatory parameters are experimentally available and differ between the two promoters due to point mutations (Table 1), we addressed the question, whether the modeling could help understanding these distinct roles.

The main known difference between these genes is the level of induction by pectin. They exhibit a comparable expression level in the presence of PGA, whereas *pelE* still has a significant level of expression in absence of inducer, whereas that of *pelD* is undetectable (10, 27). In the modeling, any observed difference in the expression results primarily from the respective binding affinities of the two promoters with the KdgR repressor, which differ by a factor of 10 (0.9 nM for *pelD* and 10 nM for *pelE* promoter). With a cellular concentration of KdgR in the micromolar range as measured *in vivo* (12), both promoters are mostly bound by the repressor in the absence of PGA in the medium (Fig. 5*I*); however, because of the affinity difference, the unbinding events are (relatively) far more frequent at the *pelE* than *pelD* promoter, hence the relatively higher basal expression level of the former (Fig. 5*J*). Still in the glucose medium, in midexponential phase, fast growth results in KdgR dilution by cell divisions, thus weakening its repressive effect on *pelE* and favoring an expression peak before transition time (Fig. 5*I*). In the presence of PGA, most of KdgR is bound by KDG in the midexponential phase, which reduces the concentration of free KdgR below the levels required for the binding of both promoters and fully activates their expression (Fig. 5*C*). Altogether, the very different induction rates of the two genes by PGA (Table 1) thus result from their different basal activity in absence of the inducer.

Activation by CRP is also different at the two promoters, but the binding affinities differ only by a factor of 2 (Table 1 and Fig. 5*D*), resulting in the profiles of similar shape characterized by a sharp peak at transition to stationary phase; however, this peak has a much stronger magnitude for *pelD* due to the three-fold higher activation factor of CRP compared with *pelE* (15).

Altogether, the combined regulation by KdgR and CRP induces both genes almost simultaneously, as observed, short before transition, that is, when induction by KDG is still significant although the exhaustion of glucose has already triggered the induction by cAMP. The reader may note a small discrepancy in the predicted expression of *pelE* (Fig. 5*F*), which occurs 1 to 2 h too late compared with experimental data. Such a deviation was not unexpected though, because *pel* promoters are sensitive to many regulators disregarded in our simplified model (8), as said above. In particular, we note that *pelE* is one of the *D. dadantii* promoters most strongly repressed by DNA supercoiling relaxation (17, 27), which occurs precisely at the transition to stationary phase (28) and is thus expected to displace the *pelE* expression peak toward earlier time, as observed.

### Early induction of *pel* genes by addition of cAMP

We wished to test the key prediction of the proposed model, that is, the role of cAMP-CRP as the key signal controlling the timing of *pel* expression, in conjunction with KDG-KdgR. We therefore ran an additional experiment where cAMP is added in the culture medium from the beginning. Based on the regulatory signals obtained in Figure 5, *C* and *E*, we expected the *pel* expression peaks to occur much earlier than in the absence of external cAMP, and more precisely, after around 5 h of growth, that is, at the timepoint where KDG has started to be synthesized from the early degradation of pectin and both regulatory signals are “on”. This prediction is in stark contrast with that of the earlier model of *pel* expression dynamics (12), where the *pel* expression time is defined by the sole feedback loop of pectin degradation and should be insensitive to the presence of cAMP (even considering an up-regulation of *pel* promoters by cAMP-CRP, because the rate limiting step in that model is not the activity of Pel enzymes but the slow subsequent import of oligogalacturonides).

**Figure 6:**
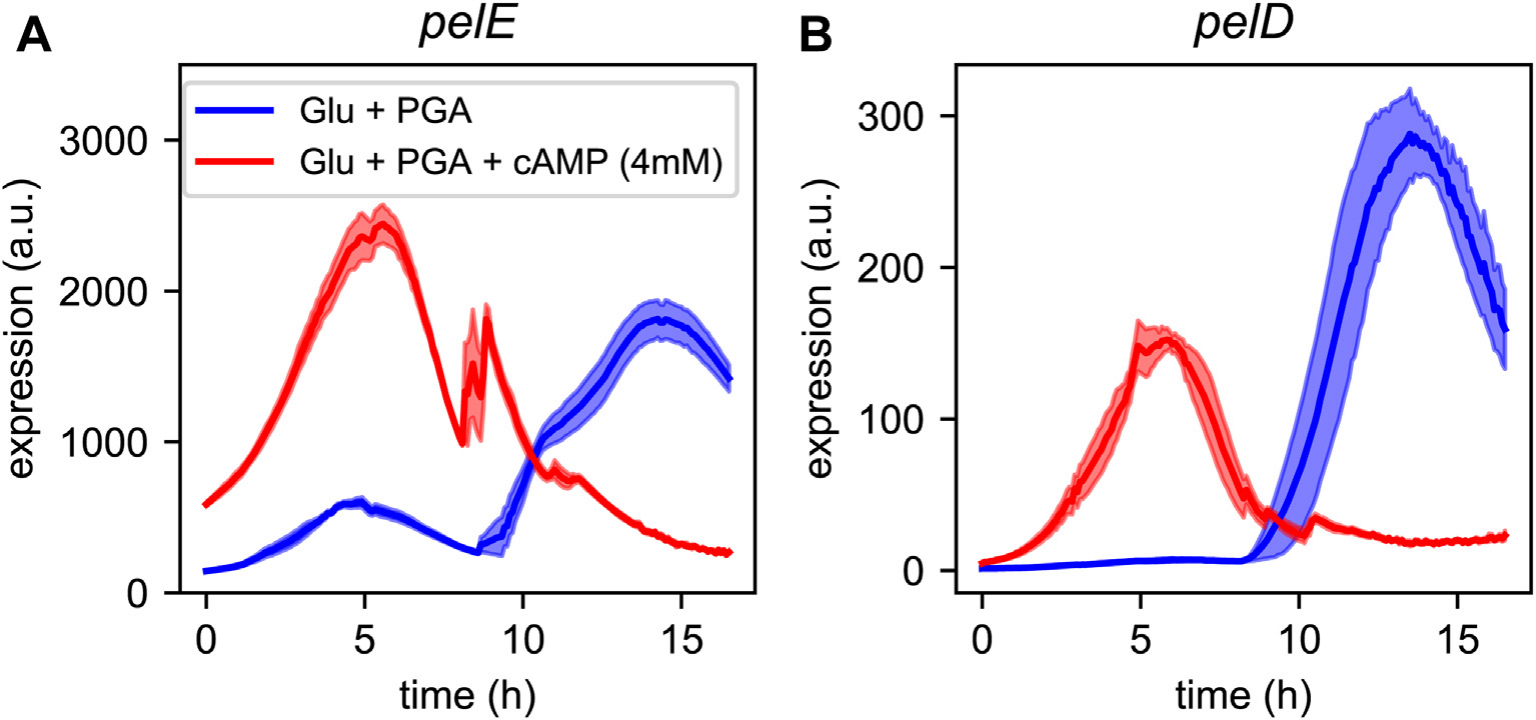
**cAMP induces an early expression of*pel*genes.** Expression profiles of the *pelE* (*A*) and *pelD* (*B*) promoters, measured with a luciferase reporter in a microplate reader, in minimal medium supplemented with glucose+PGA, in the absence (*blue*) or presence (*red*) of cAMP (4 mM, added at the beginning of the experiment). Each curve is the average of four replicates, and the shaded areas represent one SD. Pel, endo-pectate lyase; PGA, polygalacturonate.

The expression profiles of *pelD* and *pelE* were measured in real time in a microplate reader, by means of a chromosome-borne luciferase reporter system (see Experimental procedures). The reporter substrate, luciferin, was added in the culture medium, in the absence or presence of cAMP. The results are visible in Figure 6. In the absence of cAMP, the expression peaks of both *pel* genes occur after around 13 h of growth, that is, close to the transition to stationary phase (although the medium composition is the same as in batch cultures except for luciferin, bacterial growth is slower in microplates, see growth curves in Fig. S5). In the presence of cAMP, both peaks occur much earlier, precisely at the expected timepoint (after 5 h of growth). Although the exact timepoint of KDG accumulation might be slightly delayed in the microplate system, these data thus strikingly confirm the prediction of the model. It might be noted that *pelE* also exhibits a slight secondary peak at the same timepoint in the absence of cAMP (blue curve), which also matches the secondary peak observed in batch cultures (Fig. 5*F*).

To test if carbon catabolite repression also occurs in very different growth conditions, we ran an additional batch experiment in LB (rich) medium supplemented with PGA, where growth is faster than in minimal medium (Fig. S6). In the absence of added glucose, the Pel activity increased after around 5 h and remained constant until reaching the stationary phase (green curve). When glucose was added (at a higher concentration of 0.4%, resulting in a strong level of catabolic repression), the production of Pel enzymes was drastically reduced, and the recorded levels remained at insignificant levels until the end of the experiment (blue curve). Conversely, adding cAMP in the medium (red curve) resulted in an approximately 3-fold increase in Pel activity along growth. Although these data lack the time resolution required to analyze the dynamics of the system, the observed relative levels of Pel production confirm the validity of the proposed model of carbon catabolite repression involving cAMP in this very different growth condition.

## Discussion

### Regulation of *pel* gene expression during plant infection

The presented model quantitatively reproduces the expression dynamics of *pel* genes and pectin degradation in a culture medium composed of a combination of glucose and pectin. One of the most surprising features of our observations is that, in these conditions, most Pel enzymes are produced *after* pectin has been entirely degraded, and they remain therefore unused. This behavior might be rationalized if we consider the differences between the growth conditions in our stable culture and in the course of plant infection, which is the main context of evolutionary selection.

**Figure 7:**
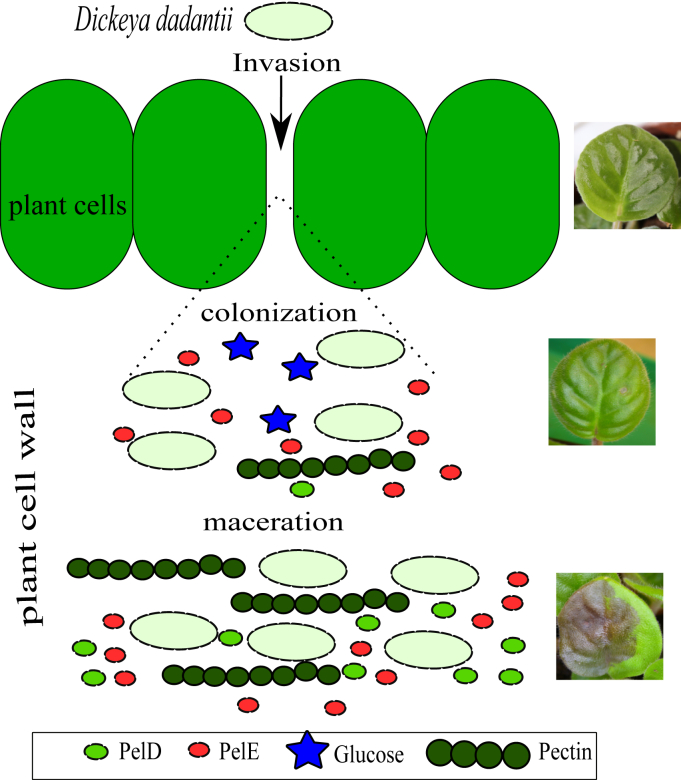
**Schematic depiction of the successive steps of plant infection by *Dickeya dadantii*: invasion, colonization, and maceration (*top* to *bottom*).** During colonization (asymptomatic), the bacteria grow on glucose in the plant apoplast. Maceration is characterized by a sudden and massive production of Pels responsible for pectin degradation (visible as *soft rot*). CRP, cAMP receptor protein; KDG, 2-keto-3-deoxygluconate; Pel, endo-pectate lyase.

The availability of carbon sources in this process is summarized in Figure 7. The timeliness and efficiency of the drastic transition between colonization and maceration (where Pels are massively produced) are critical for the success of the infection, because oligogalacturonates, the product of Pel enzymatic activity, are the main signal inducing the plant defense reactions. Any production of Pels in significant amounts thus triggers a survival race between the plant and the bacteria; if this event occurs before reaching sufficient bacterial density, or if the production boost is not sufficient, bacterial cells will ultimately be destroyed. Does our model provide insights into the regulation events associated to this intrinsically dynamical process?

Our culture conditions aim at mimicking those encountered during the colonization stage, with bacteria mostly growing on simple sugars, and a level of Pel production remaining sufficiently low to avoid a significant reaction from the plant. Crucially, we have shown that the transition to a high Pel production regime (and thus to the symptomatic phase) is triggered not by an initial event of pectin degradation as suggested earlier (12), but rather by the exhaustion of glucose in the medium (and possibly of other simple sugars in the plant), resulting in a peak of cAMP and *pel* expression boost. The expression of key virulence factors is thus intrinsically related to a metabolic switch of the bacteria.

In the second stage of infection, however, the conditions in the plant deviate from those of our stable culture medium. Although in the latter, as said above, the low amount of available pectin was already entirely consumed way before the main peak of *pel* expression, in the plant, the production of Pels induces a sustained supply of additional pectin as the soft rot propagates in the plant tissues, more similar to the conditions of a continuous bioreactor (29). Based on our modeling, this difference should have a significant regulatory consequence for *pel* genes.

Here, the activating effects of KDG and cAMP were essentially separated in time (6 h and 10 h, Fig. 5, *C* and *E*), with KDG activation having already dropped sharply at the time of *pel* expression (8 h). In contrast, with a sustained supply of pectin, KdgR would remain unbound whereas cAMP peaks, and the simultaneous activation by the two pathways would then induce a considerably stronger expression boost, as required for an efficient infection. In addition, the different regulatory properties of *pelE* and *pelD* imply that they would be differently affected by this boost. On the one hand, we expect *pelE* to keep playing a crucial role as an inducer of the Pel/KDG feedback loop, because of its higher basal expression level in the absence of pectin compared with *pelD* (26). On the other hand, whereas in our data, the two genes have a comparable maximal expression level (Fig. 5*F*), we expect *pelD* to be expressed at a much (approximately three-fold) higher level than *pelE* in the case of a simultaneous activation by both pathways, because it is much more inducible by CRP (Fig. 5*E*) and should thus play a major role in the massive production of Pels during the maceration phase. In summary, in contrast to our culture conditions where *pelE* plays a predominant role, we expect the two genes to play a qualitatively distinct but equally crucial role in the plant, which is fully consistent with the observation that both *pelD* and *pelE* mutants exhibit phenotypes with strongly reduced virulence (25). The coexistence of these two genes may thus provide a significant evolutionary advantage, possibly explaining their conservation in several *Dickeya* species (*D. dadantii*, *Dickeya solani*, *Dickeya zeae*, and *Dickeya dianthicola*) compared with close relatives (*e.g.*, *Dickeya paradisiaca*) and their ancestor (10).

### Carbon catabolite repression in *D. dadantii*

The competitive uptake of either carbon source presents many similarities, but also interesting differences with the classical example of carbon catabolite repression involved in the glucose/lactose switch of *E. coli* (13, 14).

The first apparent and previously identified difference is the monotonous aspect of the growth curve in the mixed medium (Fig. 1*A*), which contrasts with the diauxic growth of *E. coli* in glucose+lactose (30, 31). Even though pectin is a complex polysaccharide, the consumption of which depends on the production, export and activity of many degrading enzymes, this more continuous pattern previously suggested that pectin consumption starts while glucose is still available, whereas *E. coli* uses lactose only after glucose exhaustion (30). Our KDG quantification data confirm this hypothesis and even demonstrate that in our culture conditions, pectin is already converted into KDG after 5 h, that is, only half the entire duration of growth. Can we relate this behavior to the underlying regulatory properties of the *pel* genes, and extend the comparison to those of the *lac* operon in *E. coli*?

**Figure 8:**
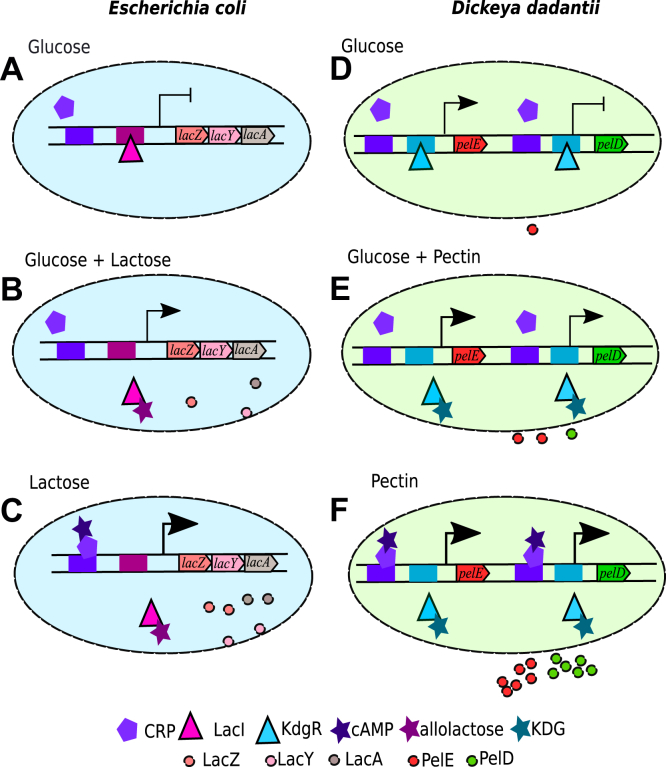
**Schematic depiction of the*lac*operon regulation in*Escherichia coli*(*left*) and*pelED*regulation in*Dickeya dadantii*(*right*), in the presence of glucose, lactose/pectin, or both sources of carbon, illustrating carbon catabolite repression in both systems.***A–D,* glucose; *C–F*, lactose/pectin; *B–E*, both sources of carbon. The promoters are shown as *arrows*, with expression levels indicated by *arrowhead* sizes (and full repression by ⊣). The genes are indicated along the DNA double helix, and the regulator binding sites are shown as *colored boxes*. Pel, endo-pectate lyase.

Figure 8 gives a schematic depiction of the regulatory systems of these operons controlling the catabolism of the secondary sugar sources in both species (lactose and pectin, respectively). These systems present a conspicuous similarity, with an activation by cAMP-CRP and repression by a pathway-specific regulator, LacI and KdgR, respectively, which can be driven away by the degradation product of the sugar, allolactose or KDG respectively. On the other hand, the modes of action of the enzymes involved in the two catabolic pathways are very different, because pectin is a polysaccharide degraded outside the cells by Pels, whereas lactose is a disaccharide and is imported when LacY is present at the cell membrane (31).

In the presence of glucose alone (top), the activator CRP is unbound, but the repressor inhibits the expression of *pel*/*lac* genes. In *D. dadantii*, however, the presence of a promoter with low repressor affinity (*pelE*) results in a low basal expression and a less drastic repressive effect. When both sources of carbon are present simultaneously (middle), a moderate level of expression can be sustained by the release of the repressor. When only the secondary source is present (bottom), the expression is boosted by the binding of cAMP-CRP.

As visible in Figure 8, among the two *pel* genes considered in this study, *pelD* is the one with a regulatory system most comparable with the *lac* promoter, exhibiting a strict dependence on the availability of the secondary carbon source. In this figure showing a stationary behavior in each medium, the difference between the two *pel* genes appears quite limited, however, and their coexistence would probably not constitute a key advantage if the bacterium lived in a stable or slowly changing environment, as nonpathogenic *E. coli* does most of the time. On the other hand, when both sources of carbon are present (middle panel), the depicted partial expression requires an initial expression event, to start producing allolactose/KDG and sustain subsequent transcription. In *E. coli*, because of the absence of a significant basal level of *lac* expression, this initiation step may be very long, partly explaining why glucose is entirely consumed first (diauxic growth). In *D. dadantii*, the basal expression of *pelE* drastically reduces this initiation time, facilitating the switch in carbon source and resulting in continuous growth. Interestingly, measurements in a *pelE* mutant strain indeed exhibited a severe delay in the expression of *pel* genes, due to a much longer initiation step of the pectin degradation feedback loop (10). As discussed in the previous section, the evolutionary advantage of having coexisting *pelD* and *pelE* genes is thus probably intimately related to the dynamical requirements of *pel* expression in the context of pathogenesis. This expression is part of a race with the host’s defense systems where time plays a prominent role compared with the requirements of nonpathogenic bacteria, which are more specifically selected for resource optimization and where the existence of two *pelE/D* genes with specialized roles (rapidity of the switch *versus* expression boost) might be key to the success of *D. dadantii*.

### Relevance of the model in various bacterial systems

Microbial metabolism of plant polysaccharides is an important part of environmental carbon cycling, human nutrition, and industrial processes based on biomass bioconversion.

The two regulatory pathways that were included in our model are present in most pectinolytic bacteria of the genera *Dickeya* and *Pectobacterium* responsible for plant soft rot disease. Based on protein homology, they might be also partly present in several other species found in plants or in the digestive tract of animals, including from genera *Xanthomonas*, *Yersinia*, *Pseudomonas*, *Bacillus*, and *Streptomyces* (32).

On the other hand, the regulator KdgR is conserved in many more enterobacteria where it controls the catabolism of uronic acids, in particular, in *E. coli*, *S. enterica*, and *Yersinia enterocolitica*, which are found in various ecological niches associated with the human body and free-living in the environment. These bacteria lack most extracellular pectinases and the Out transport system necessary for their secretion, as well as other enzymes responsible for the degradation of pectin derivatives (33, 34), and their ability to catabolize pectin derivatives thus depends on the latters’ prior degradation into uronic acids by other bacteria or by the plant. In *Y. enterocolitica*, the uptake of oligogalacturonides was shown to be controlled by a regulatory feedback loop involving KdgR and may thus follow a similar model as presented here, albeit devoid of the initial steps of pectin depolymerization (35). *S. enterica* was found to be highly adapted to plant soft rot lesions induced by *D. dadantii*, where it feeds on uronic acids resulting from the activity of Pel enzymes, in anaerobic conditions similar to those of the animal intestine, which may be responsible for its frequent presence in harvests (36). KdgR is also present in the bacteroidetes inhabiting the human gut which have evolved under intense pressure to use complex carbohydrates, primarily plant cell wall glycans from our diets (37). In this context, the human colonic microbiota is able to simultaneously degrade complex mixtures of polymerized carbon sources, thanks to different bacterial species that have varied substrate preferences and are involved in the sequential usage of some carbohydrates. These different bacterial species form distinct patterns of metabolic end products, and catabolite regulatory mechanisms ultimately affect the types and amounts of these substances that they can produce from individual substrates. The modeling of such interactions between species is more complex than the system presented here, because they involve competitive growth and various carbon sources, but our modeling of the dynamics of pectin depolymerization constitutes an important step for that purpose.

Notably, the presence of KdgR in *E. coli* has been exploited in industrial processes based on the bioconversion of pectin-rich biomass for the production of fuel ethanol after the addition of the *Dickeya pelE* and *out* genes in the ethanologenic *E. coli* strain (38). Although the detailed kinetic parameters of the employed pathway likely deviate from those of *D. dadantii*, a quantitative understanding of the dynamic interplay between pectin uptake and bacterial growth is an important step for the optimization of such processes.

## Experimental procedures

### Bacterial strain and culture conditions

*D. dadantii* strain 3937 was used for all experiments described. The cultures were grown at 30 °C in M63 minimal salt medium (39), supplemented with 0.2% (w/v) glucose as primary carbon source and 0.4% (w/v) PGA for pectin degradation studies. Liquid cultures were grown in a shaking incubator (125 r.p.m.).

### Gene activity assays

*pelE/D* expression were measured by qRT-PCR (Figs. 1*C* and 5), as described in (12).

Pectate lyase enzymatic activity assays were performed on toluenized cell extracts in 1 ml cell culture harvested at predetermined time points along the growth of the bacteria. The degradation of PGA by pectate lyases is monitored by absorption spectrometry of unsaturated oligogalacturonides at 230 nm (40). Pel activity is expressed as μmol of unsaturated products liberated per minute and per ml of enzymatic extract, as described in (12). All experiments were carried with two biological replicates.

The effect of cAMP on *pelE/D* expression (Fig. 6) was measured in real time in a microplate reader (TECAN), using mutant strains carrying a luciferase reporter gene controlled by the *pelE/D* native promoter (inserted in the chromosome between *pelA* and *pelE* genes). The employed protocol is described in (41). Shortly, the microplates were prepared with minimal medium containing glucose+PGA (at the same concentrations as in batch cultures) and the substrate luciferin, in the presence or absence of 4 mM cAMP, and inoculated with either mutant strain (at absorbance = 0.1). Optical densities and luminescence were recorded every 5 min (see raw datapoints in Fig. S5), and the expression was defined as the ratio of luminescence/absorbance.

### Bacterial samples preparation for KDG and cAMP quantification

The bacterial culture samples were harvested at predetermined time points after measuring the absorbance, centrifuged at 8000 rpm for 6 min to separate cells from the supernatant medium. Bacterial pellet was freeze dried with liquid nitrogen for storing. The cells and medium samples were stored separately at −80 °C. To process for quantification, the cell pellet was resuspended in 10 ml cold solution of 2.5% (v/v) TFA and 50% (v/v) acetonitrile. The dissolved lysed pellet was maintained at −80 °C for 15 min and thawed at room temperature. The solution was then centrifuged at 20,000*g* for 5 min. The clear supernatant containing the metabolites (10 ml) was separated from the pellet and then lyophilized overnight. The dry powder was dissolved in 1 ml of 0.1 M HCl and centrifuged again to remove particles. The culture medium is directly made up to 0.1 M HCl with 1 M HCl. The measurements were carried in two biological replicates in glucose+PGA medium (and one in the glucose control medium).

### KDG and cAMP quantification using HPLC

KDG metabolite was quantified using a novel HPLC technique developed inhouse, as described in (16). The carbonyl group was derivatized with o-phenylenediamine and eluted with a buffer consisting of 0.005% TFA and 60% acetonitrile.

cAMP metabolite was quantified by HPLC after derivatization with 2-chloroacetaldehyde (20) (Fig. S2). Bacterial cell and extracellular medium sample extracts are suspended in 0.1 M HCl. Equal amounts of sample in 0.1 M HCl and 1 M sodium acetate are mixed and 10% v/v of 2-chloroacetaldehyde solution is added to the mixture and incubated at 80 °C for 30 min with continuous shaking (42). The appropriate amounts of incubated sample are injected onto an Uptisphere C18 column (Interchim) for separation of the purine derivative adducts. Buffer A was composed of 0.005% TFA in water and Buffer B of 100% methanol. The flow rate was set-up at 35 °C and run at 1 ml/min using the following gradient: initial 90% A, 10% B; linear gradient from 0 to 20 min up to 70% A, 30% B; 22 min 100% B; 25 min 25% B, then reequilibration to initial condition from 28 to 30 min (see the profile in Fig. S3). The mean retention time for cAMP was between 13.04 and 13.10 min. The associated peak is well-separated from neighbor peaks characteristic of other compounds of similar structure (Fig. S3*B*) and can be further distinguished by its characteristic absorption/emission spectrum identical to that of the purified molecule (data not shown). The quantification is performed from a standard curve (Fig. S4). The raw datapoints from extracellular extracts (Fig. 4) were subjected to spline interpolation of degree 3, as well as the growth curve (Fig. 1*A*), and intracellular concentrations were inferred from those using a kinetic model of cAMP import/export adapted from *E. coli* (21), with adjusted import/export rates (details in Supplementary Information, p. S4).

### Model

The system dynamics is simulated with a set of deterministic ordinary differential equations relating the concentrations of all considered species (see Fig. 2 and Supplementary Information). Promoter activities follow a thermodynamic model of transcription (24), involving the activator cAMP-CRP and repressor KdgR (Table 1). All the enzymatic reactions are treated with Michaelis–Menten kinetics. The list of model parameters is provided in Table S2. All thermodynamic and regulatory parameters were obtained experimentally (12, 15, 18) and most kinetic parameters. The five remaining parameters (Table S3) were numerically estimated by fitting a set of observed quantities (*pel* expression peak time and ratio in the presence and absence of PGA, KDG concentration peak time, and magnitude) using the truncated Newton method. The dynamical system was simulated with the Euler algorithm, using a constant timestep of 0.3 min ensuring numerical stability. All the data analyses and computations were carried in Python using NumPy, Scipy, and Pandas libraries; the simulation code is available upon request.

## Data availability

All presented data are contained in the article. The software code is available upon request (S. M.).

## Supporting information

This article contains supporting information.

## Conflict of interest

The authors declare that they have no conflicts of interest with the contents of this article.
