# Supplementary material for: Carbon catabolite repression in pectin digestion by the phytopathogen Dickeya dadantii
Source: J Biol Chem. 2021 Nov 23;298(1):101446. doi: 10.1016/j.jbc.2021.101446 (PMC8688573; doi:10.1016/j.jbc.2021.101446)
Supplement: Supplemental Figures S1–S6 and Tables S1–S4 [file mmc1.pdf]

Carbon catabolite repression in pectin digestion by the phytopathogen *Dickeya dadantii*

Shiny Martis B., Michel Droux, Sylvie Reverchon, William Nasser and Sam Meyer

Supplementary Information

Transcriptional regulatory model for pel expression

The transcriptional regulatory model for *pel* expression and PGA degradation is defined by the set of following ordinary differential equations, extending a previous model of the KDG/KdgR pathway (12):

$$\frac{d\rho}{dt} = \sigma * \rho * \left(1 - \frac{\rho}{\rho_s}\right) \tag{1}$$

$$\frac{dy}{dt} = \beta_2 - \left(\alpha_2 + \frac{\rho^o}{\rho}\right) * y \tag{2}$$

$$\frac{dX_E}{dt} = \beta_{1E} * \frac{\rho}{1-\rho} * \frac{K_{d6E}}{K_{d6E}+y_d} * \frac{K_{aE}+CAP*f_E}{K_{aE}+CAP} - \left(\alpha_1 * \frac{K_m}{K_m+s} * X_E\right) \tag{3}$$

$$\frac{dX_D}{dt} = \beta_{1D} * \frac{\rho}{1-\rho} * \frac{K_{d6D}}{K_{d6D}+y_d} * \frac{K_{aD}+CAP*f_D}{K_{aD}+CAP} - \left(\alpha_1 * \frac{K_m}{K_m+s} * X_D\right) \tag{4}$$

$$\frac{ds}{dt} = -\epsilon * k_{cat} * \left(X_E + X_D\right) * \frac{s}{s+K_m} \tag{5}$$

$$\frac{dz}{dt} = 2\epsilon * k_{cat} * \left(X_E + X_D\right) * \frac{s}{s+K_m} - \gamma * \rho * z \tag{6}$$

$$\frac{dw}{dt} = \gamma * (1 - \rho) * z - k_5 * \left(\frac{w}{w+K_w}\right) + \frac{\rho^o}{\rho} * w \tag{7}$$

$$y = 2y_d * \left(1 + \frac{w^2}{k_{d3}^2}\right) \tag{8}$$

$$CAP = \frac{[cAMP]}{[cAMP]+K_{cAMP}} * \frac{CRP}{2} \tag{9}$$

Supplementary Table S1. List of variables in the dynamical system

Variables	Description
ρ	Bacterial volume fraction
y	Total intracellular concentration of KdgR monomer
X_E	Concentration of extracellular PelE enzyme
X_D	Concentration of extracellular PelD enzyme
s	Concentration of extracellular PGA substrate
z	Concentration of extracellular UGA (end product of PGA degradation)
w	Concentration of KDG
y_d	Concentration of free KdgR dimer
CAP	Concentration of CRP.cAMP complex, inferred from the measured values of cAMP concentration (Fig. 1C)

Note that each KdgR monomer is able to bind a KDG molecule (12), whereas we consider only the dominant form of the cAMP-CRP complex involving a CRP dimer and one molecule of cAMP (30) (Eq. 9).

Supplementary Table S2. Parameters of the model with fixed value

	Description	Value	Unit	Source
α_1	First-order degradation rate coefficient of PelE protein	0.0494	h^{-1}	(12)
α_2	First-order degradation rate coefficient of KdgR protein	0.0347	h^{-1}	(12)
β_2	Maximum rate of synthesis of KdgR	0.3	$\mu M.h^{-1}$	(12)
ε	Ratio of the enzymatic rate constant of Pel reactions in the culture medium and buffer	1/3		(12)
K_{d6E}	Equilibrium dissociation constant of the KdgR dimer at the *pelE* promoter	0.01	μM	(15,18)
K_{d6D}	Equilibrium dissociation constant of the KdgR dimer at the *pelD* promoter	0.0009	μM	(15)
K_{aE}	Equilibrium dissociation constant of the cAMP-CRP complex at the *pelE* promoter	0.0006	μM	(15)
K_{aD}	Equilibrium dissociation constant of the cAMP-CRP complex at the *pelD* promoter	0.0003	μM	(15)

K_m	Michaelis-Menten constant for Pel.PGA complex	6800	μM	(12)
k_{cat}	Rate constant of the enzymatic Pel reactions	3600000	h^{-1}	(12)
CRP	Effective intracellular concentration of CRP monomer	0.005	μM	arb
K_{d3}	Equilibrium dissociation constant of the KdgR-KDG complex	400	μM	(11)
f_E	*pelE* activation factor by cAMP-CRP complex	60		(15)
f_D	*pelD* activation factor by cAMP-CRP complex	180		(15)
K_{cAMP}	Equilibrium dissociation constant of cAMP and CRP dimer	10	μM	(43)

Supplementary Table S3. Unknown parameters adjusted on the data

Parameter	Description	Value	Unit
γ	Cell membrane import rate	700	h^{-1}
β_{1E}	Maximum rate of synthesis of PelE	20.0	$\mu M.h^{-1}$
β_{1D}	Maximum rate of synthesis of PelD	15.0	$\mu M.h^{-1}$
k_5	Phosphorylation rate of KDG	3.0	h^{-1}
K_w	Phosphorylation constant of KDG	5000	μM

Supplementary Table S4. Growth parameters

ρ^o	Initial bacterial volume fraction
ρ_s	Maximum bacterial volume fraction
s_0	Initial substrate concentration
σ	Bacterial growth rate

In this study, the medium contains a fixed quantity of glucose, and the effect of varying PGA is studied. The dependence of parameters on bacterial growth on the initial amount of PGA substrate s_0 was obtained to be linear (12) and given by

$$\sigma = 0.59 + 0.56\, s_o \tag{10}$$

$$\rho_s = (1.75 + 1.71\, s_o) \times 10^{-3} \tag{11}$$

Kinetic model of cAMP import/export

A direct quantification of intracellular cAMP concentrations is challenging because of the fast import/export dynamics of cAMP (21), resulting in a lack of reproducibility depending on the protocol and duration of the experiment. Following an existing procedure, we therefore measured the concentrations in the extracellular medium and inferred the intracellular concentrations using a kinetic import/export model (21). The effective equation relating the intracellular/extracellular concentration is:

$$cAMP_{in} = \frac{C_1}{OD(t)} * \frac{d(cAMP_{out})}{dt} + C_2 * cAMP_{out} \qquad (12)$$

where C1 and C2 are two constants related to the total culture volume, cell volume, and cAMP import and export rates (21). The extracellular cAMP concentrations measured in our conditions are significantly higher than those measured in the original experiment with *E. coli* in microplates, but the *in vitro* regulatory properties of the purified CRP proteins from both species are indistinguishable on *pel* genes (15). We assumed that the kinetic import/export parameters could differ because of differences in species or growth conditions (batch culture vs microplates), and we adjusted their values based on those estimated in *E. coli*, so that the peak magnitude in internal cAMP concentration approximately matches the affinity of the metabolite for CRP (10 µM, dashed horizontal line in Fig. 1C) (43), and thus triggers the regulatory action of the latter, as expected for this signaling molecule. As a result, the intracellular concentrations are given by:

$$cAMP_{in} = \frac{22}{OD} * \frac{d(cAMP_{out})}{dt} + 0.20 * cAMP_{out} \qquad (13)$$

and are computed after a spline interpolation of the extracellular measures to reduce noise. We note that the absolute magnitude (but not the timing) of the intracellular concentration peak depends on the adjusted import/export parameters and thus remains imprecise, but these quantitative variations are buffered in the resulting cAMP-CRP concentration pattern, and we checked that limited parameter variations do not change the timing of cAMP activation.

Supplementary Figures

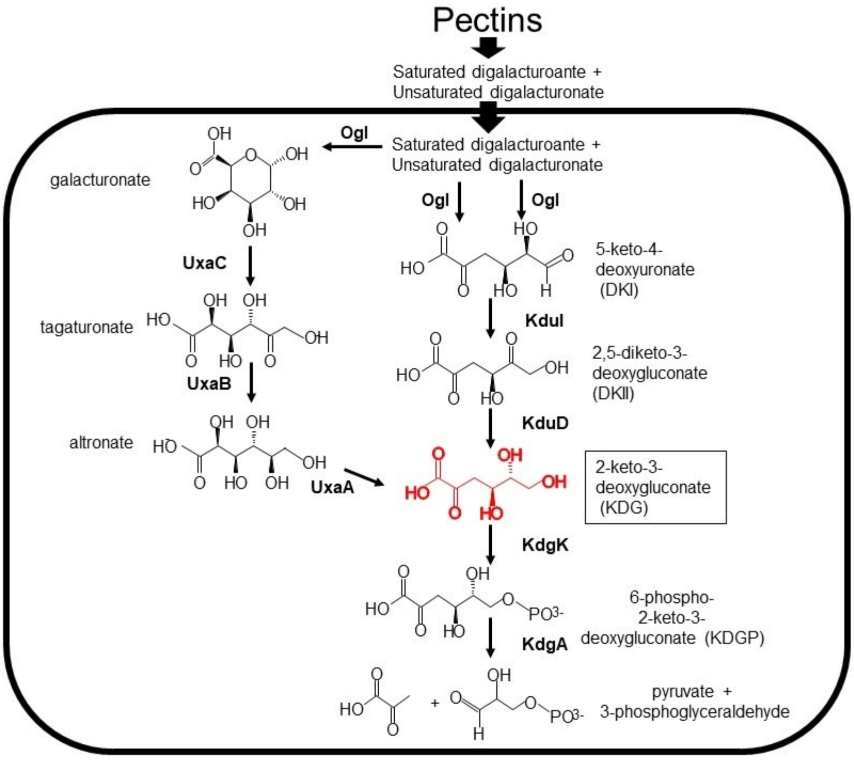

Supplementary Figure S1. Detailed pectin degradation pathway

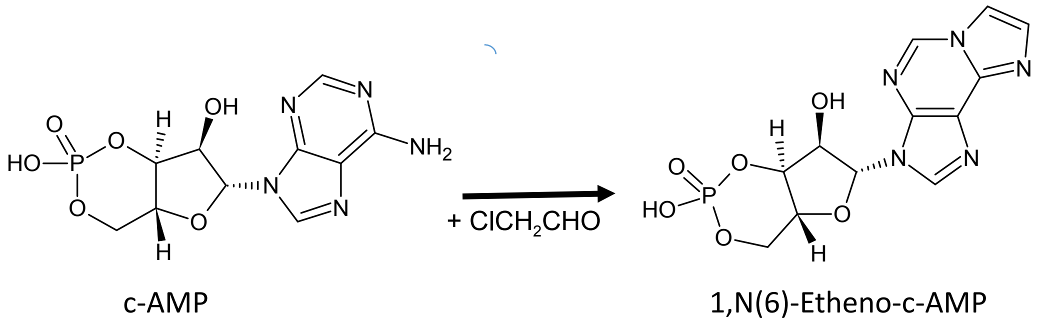

Supplementary Figure S2. Derivatization of cAMP with 2-chloroacetaldehyde

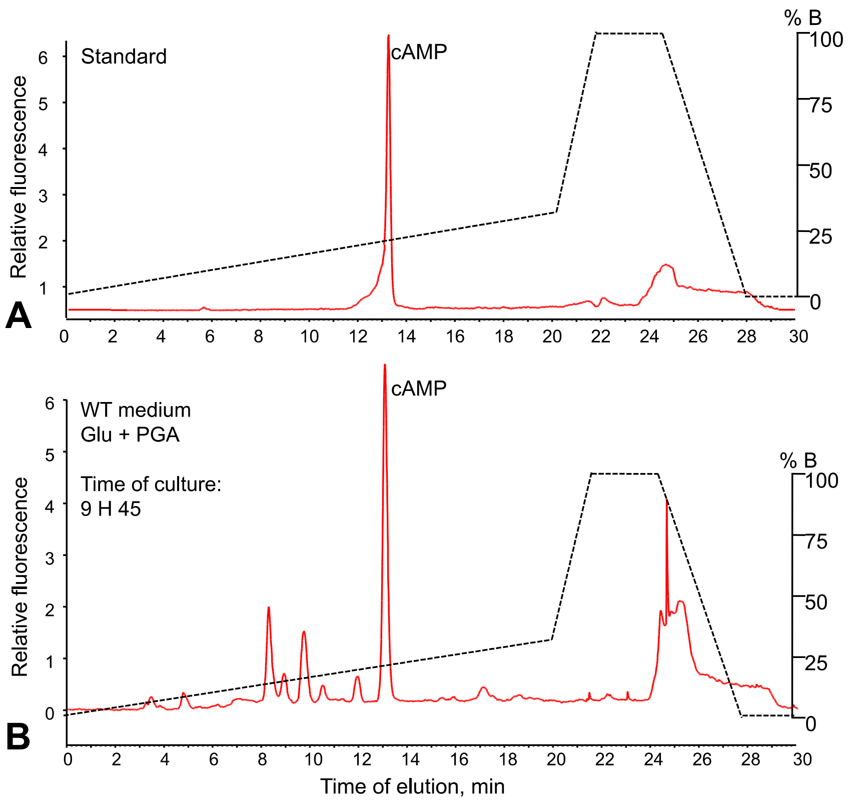

Supplementary Figure S3. Elution profile of cAMP by fluorescence detection at 418 nm after specific excitation at 278 nm, in a sample containing purified cAMP (A) or extracellular culture extracts in M63 Glucose+PGA medium (B). The fluorescence signal is shown in red (left scale), and the proportion of methanol (vs trifluoroacetic acid) in the elution buffer is shown as a dashed black line (right scale).

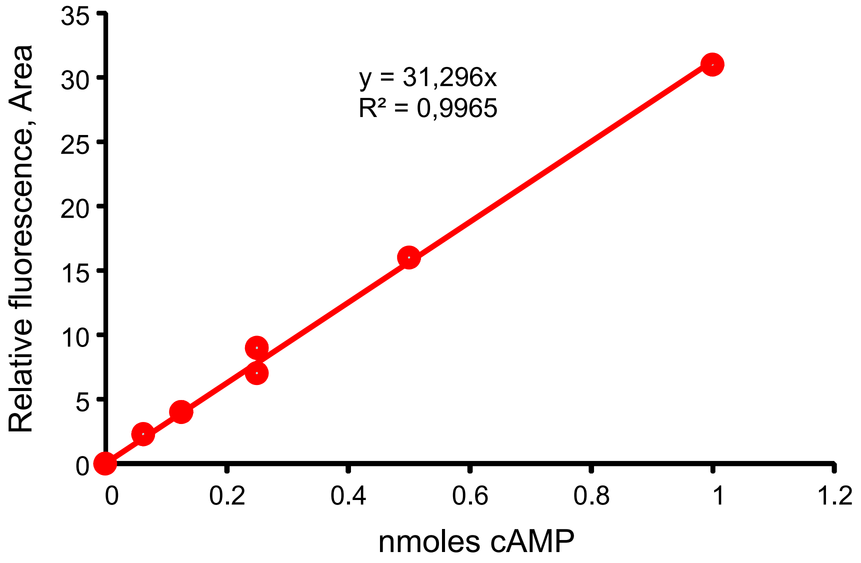

Supplementary Figure S4. Standard curve of cAMP titration.

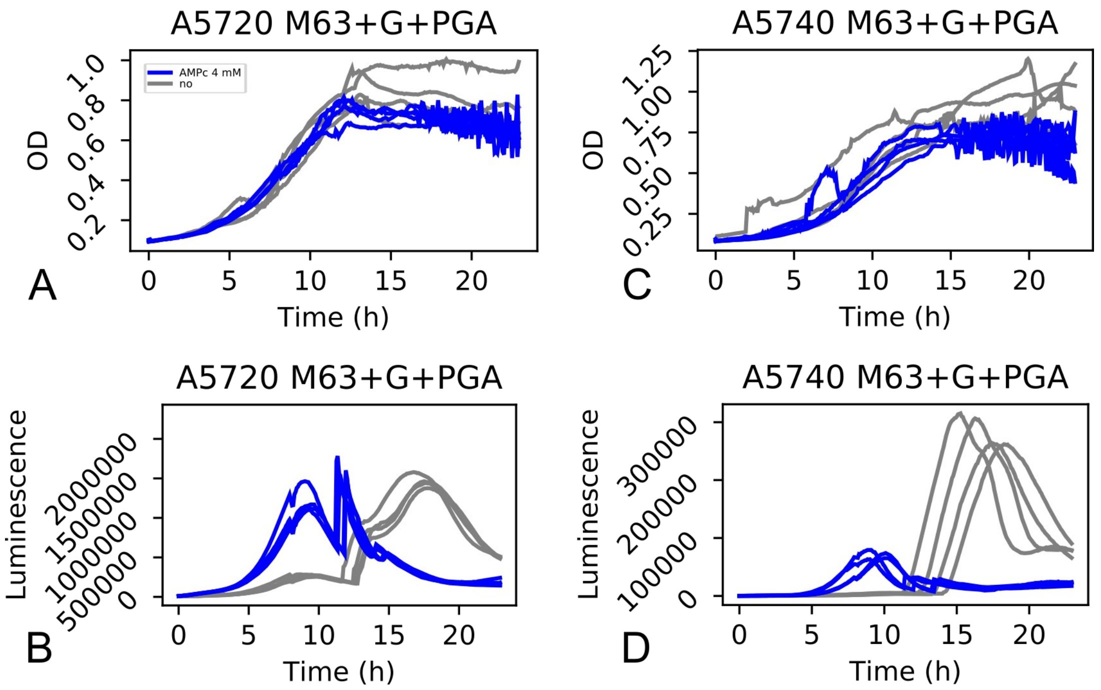

Supplementary Figure S5. Raw datapoints of *D. dadantii* strains containing a luciferase reporter controlled by *pelE/D* in presence and absence of cAMP, as recorded in a microplate reader. (A) Optical densities of the A5720 strain (*pelE*::lux); (B) Luminescence of the A5720 strain; (C) Optical densities of the A5740 strain (*pelD*::lux); (B) Luminescence of the A5740 strain. Data obtained in presence (resp. absence) of cAMP (4 mM) are shown in blue (resp. gray). Each trace corresponds to a well of the microplate, and all conditions were analysed in four replicates. The frequent irregularities of optical density curves are due to heterogeneous hits of laser beams in presence of floculation.

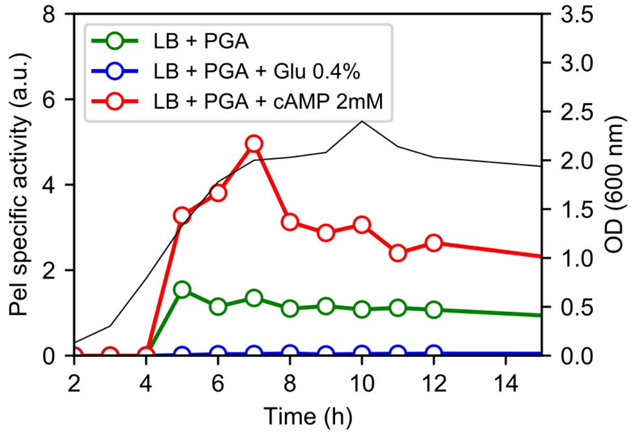

Supplementary Figure S6. Pel activity measured along bacterial growth in LB medium + PGA (green), supplemented with glucose (blue curve, note that the concentration of 0.4% is twice higher than that used in the main experiments, resulting in a strong level of catabolic repression) or cAMP (2 mM, red curve).
